# Two Decades of Desiccation Biology: A Systematic Review of the Best Studied Angiosperm Resurrection Plants

**DOI:** 10.3390/plants10122784

**Published:** 2021-12-16

**Authors:** Shandry M. Tebele, Rose A. Marks, Jill M. Farrant

**Affiliations:** 1 Department of Molecular and Cell Biology, University of Cape Town, Rondebosch, Cape Town 7701, South Africa; TBLSHA001@myuct.ac.za (S.M.T.); marksr49@gmail.com (R.A.M.); 2 Department of Horticulture, Michigan State University, East Lansing, MI 48824, USA; 3 Plant Resiliency Institute, Michigan State University, East Lansing, MI 48824, USA

**Keywords:** angiosperm resurrection plants, desiccation tolerance, omics technologies, systematic review

## Abstract

Resurrection plants have an extraordinary ability to survive extreme water loss but still revive full metabolic activity when rehydrated. These plants are useful models to understand the complex biology of vegetative desiccation tolerance. Despite extensive studies of resurrection plants, many details underlying the mechanisms of desiccation tolerance remain unexplored. To summarize the progress in resurrection plant research and identify unexplored questions, we conducted a systematic review of 15 model angiosperm resurrection plants. This systematic review provides an overview of publication trends on resurrection plants, the geographical distribution of species and studies, and the methodology used. Using the Preferred Reporting Items for Systematic reviews and Meta–Analyses protocol we surveyed all publications on resurrection plants from 2000 and 2020. This yielded 185 empirical articles that matched our selection criteria. The most investigated plants were *Craterostigma plantagineum* (17.5%), *Haberlea rhodopensis* (13.7%), *Xerophyta viscosa* (reclassified as *X. schlechteri*) (11.9%), *Myrothamnus flabellifolia* (8.5%), and *Boea hygrometrica* (8.1%), with all other species accounting for less than 8% of publications. The majority of studies have been conducted in South Africa, Bulgaria, Germany, and China, but there are contributions from across the globe. Most studies were led by researchers working within the native range of the focal species, but some international and collaborative studies were also identified. The number of annual publications fluctuated, with a large but temporary increase in 2008. Many studies have employed physiological and transcriptomic methodologies to investigate the leaves of resurrection plants, but there was a paucity of studies on roots and only one metagenomic study was recovered. Based on these findings we suggest that future research focuses on resurrection plant roots and microbiome interactions to explore microbial communities associated with these plants, and their role in vegetative desiccation tolerance.

## 1. Introduction

Even though water is essential to life, plants are often faced with shortages of this valuable resource due to their sessile nature. As a result, many have evolved sophisticated strategies for resisting, avoiding or tolerating water shortages [1,2]. One of the most successful adaptations to such extreme drought is desiccation tolerance—the ability to survive water loss to 10% relative water content (RWC), equivalent to 0.1 g H_2_O/g dry weight, and revive full metabolism when rehydrated [3]. Such plants are commonly called resurrection plants [4]. Desiccation tolerance is common in seeds, spores, and pollen, but very rare in vegetative tissues of plant, occurring in only ~240 angiosperms. Interestingly, resurrection plants are extremely diverse, representing at least 10 families [5] across both monocotyledon and dicotyledon lineages.

Resurrection plants have received growing research attention in the past 20 years and a handful of species have emerged as models for understanding desiccation tolerance. Among these are the monocot resurrection plants *Eragrostis nindensis*, *Oropetium thomaeum, Sporobolus stapfianus*, *Tripogon loliiformis, Xerophyta humilis* and Xerophyta viscosa* (multiple populations were later reclassified as *X. schlechteri*) and the dicots *Boea hygrometrica*, *Craterostigma plantagineum*, *Craterostigma pumilum*, *Craterostigma wilmsii*, *Haberlea rhodopensis*, *Lindernia brevidens*, *Myrothamnus flabellifolia*, *Ramonda serbica* and *Ramonda nathaliae*. These species are distributed across the globe, with representatives found in both the Southern and Northern hemispheres, but the highest density of resurrection plants occurs in arid tropical and subtropical regions in Africa, South America and Australia. Fewer resurrection plants are found in the Northern hemisphere, but *B. hygrometrica* and *Paraboea rufescens* occur in Asia, and *H. rhodopensis* and *Ramonda* species are endemic to Europe [6,7,8]. The ability to tolerate desiccation has enabled resurrection plants to thrive in extremely arid microclimatic conditions where other plants perish. Resurrection plants grow predominantly in sites with shallow rocky soil, high temperatures, and limited rainfall [5,9].

Resurrection plants have evolved protective mechanisms that allow them to cope with environmental stress, including a robust antioxidant defense system, sophisticated gene expression programs in which late embryogenesis abundant, heat shock proteins, and other stress responsive genes are transcribed and where necessary translated, and subtle metabolic modulations involving numerous phytohormones and phytochemicals [3,10,11,12,13]. Despite the intricacy of desiccation tolerance mechanisms in plants, scientists have made significant improvements in developing an understanding these processes.

Over the past two decades “omics” methodologies have been increasingly applied to resurrection plants to acquire insights into the biochemical processes and molecular mechanisms of desiccation tolerance. Multiple genomic, transcriptomic, proteomic, metabolomic, and physiological/biochemical studies of desiccation tolerant plants have been published in recent years. The advances in “omics” techniques enable exploration of new genes, transcripts, metabolites, proteins, and microbes that contribute to desiccation tolerance. Recently, genomic and transcriptomic analyses of *B. hygrometrica*, *C. plantagineum*, *E. nindensis*, *H. rhodopensis*, *L. brevidens*, *O. thomaeum*, *T. loliiformis*, and *X. schlechteri*, have identified gene families and transcripts involved in desiccation responses [14,15,16,17,18,19,20,21]. Metabolomic analysis of *C. plantagineum*, *L. brevidens*, *M. flabellifolia*, and *X. schlechteri* have provided insight into the central role of sugars (sucrose in particular), selected amino and organic acids, and phenolic antioxidants [22,23,24]. However, the bulk of these studies have been conducted on leaf tissues only, with the role of the root being largely ignored. Furthermore, there are limited studies addressing the microbial communities associated with resurrection plants, despite growing awareness of the importance of plant microbiota in plant host performance. Given that microbes have the potential to improve resilience to numerous biotic and abiotic stresses [25,26], it is likely that these interactions play a prominent role in desiccation tolerance.

This review describes the changing landscape of resurrection plant research over the past 20 years and identifies under–explored areas of research. We systematically summarized research efforts on resurrection plants to identify the 15 most studied resurrection plants–based on publication number—and compiled all genomic, transcriptomic, proteomic, metabolomic, metagenomic and physiological studies on these species from 2000 to 2020. In total, we summarized 185 research studies on resurrection plants to address the following questions: (i) what are the best studied resurrection plants? (ii) where are these species native to and how does that relate to where have they been studied? (iii) what types of methodologies have been applied to each species? and (iv) what are under–explored avenues of research in resurrection plants? To the best of our knowledge, this serves as the first systematic review conducted on such a diverse set of resurrection plants.

## 2. Materials and Methods

### 2.1. Literature Search and Selection Criteria

We used a multi–step protocol called Preferred Reporting Items for Systematic reviews and Meta–Analyses (PRISMA) to collate publications for this systematic review [27,28]. A literature search was conducted using EBSCOhost, Scopus, google scholar, ProQuest, PubMed, SciFinder and Europe PubMed Central (Europe PMC). The search was conducted in January 2021 using wildcards including “resurrection plants” or “desiccation tolerant plants”, “desiccation stress” or “water deficit conditions” and “dehydration and rehydration”. There were no filters applied in the search. Additional research articles that matched our search criteria were obtained from reference lists of the published reviews and original articles. This literature search generated a total of 6522 studies and all citations were exported to EndNote 20.1 for macOS.

**Figure 1:**
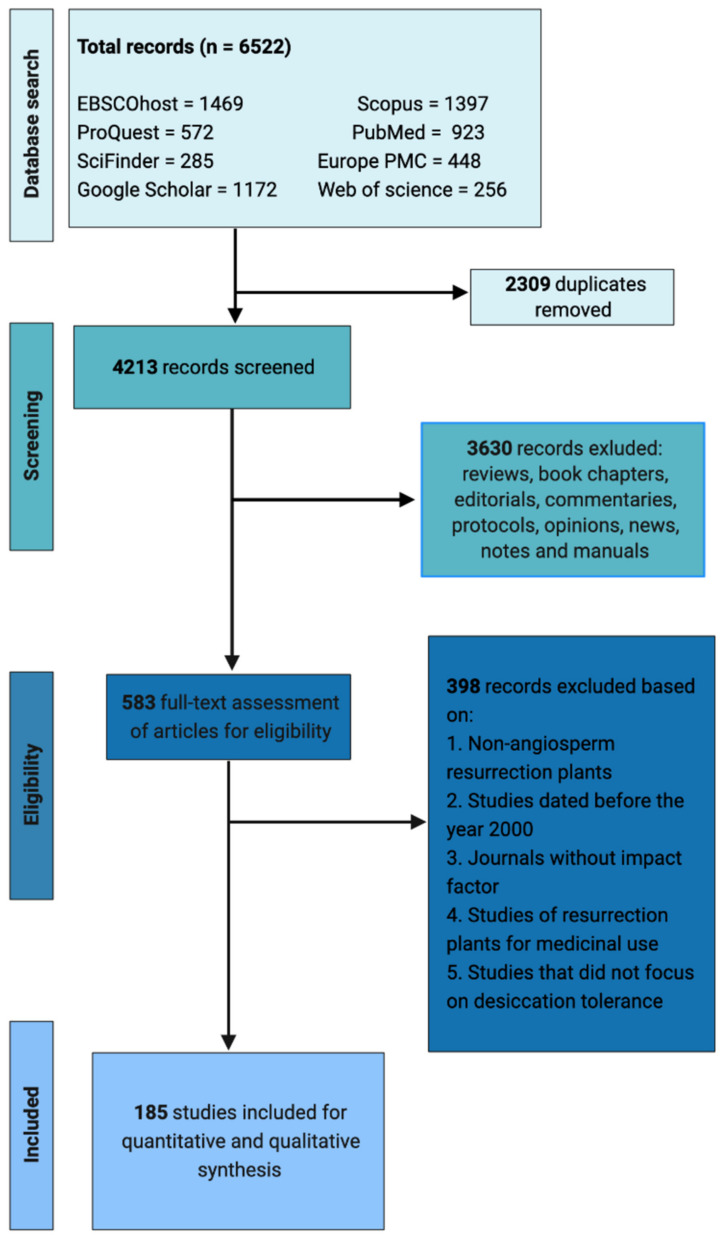
Systematic reviews and Meta–Analyses (PRISMA) flow chart outlining the exclusion and selection procedure used in the current meta-analysis with corresponding records from the database of angiosperm resurrection plants.

A screening process was conducted in the EndNote software by manually evaluating article titles and abstracts. Initial screening was performed to discard duplicates, reviews, mini–reviews, editorials, commentaries, books, book chapters, theses, dissertations, opinions, conference abstracts, presentations, meta–analyses, protocols, manuals, notes, and news. Next, selection and exclusion criteria were applied to the title and abstract of each article. Studies were excluded based on (i) research articles dated before the year 2000; (ii) scientific journals without impact factor; (iii) studies of resurrection plants for medicinal use; (iv) studies that did not focus on desiccation tolerance and defense mechanisms against abiotic stress; and (v) resurrection plants under the divisions of *Bryophyta*, *Marchantiophyta*, and *Pteridophyta*. The selection criteria for inclusion of scientific articles included (i) original full–text articles published in English from 2000 to 2020; (ii) studies involving angiosperm resurrection plants regardless of the location or country; (iii) research articles that focused on desiccation tolerance mechanisms and (iv) studies based on genomics, transcriptomics, proteomics, metabolomics, metagenomics and physiology. All records of the literature search and the number of included full–text articles were retained in accordance with the PRISMA framework (Figure 1).

### 2.2. Literature Analysis and Data Acquisition

From this set of literature, we summarized overall trends on the number and type of publications on resurrection plants from 2000 to 2020, including the number of studies conducted on each species, the country where these studies were conducted relative to the native range of the species, and the types of studies performed—with a particular focus on “omics” methodologies. Initially, we compared publication trends over time by computing the number of studies published annually from 2000 to 2020 (see Supplementary Table S1 for a detailed list of full-text surveyed articles). Next, we computed the total number of studies for each focal species. It is noteworthy that some populations of *Xerophyta viscosa* have recently been reclassified as *X. schlechteri*. In addition, the majority of papers (reference: 2, 10, 16, 18, 50, 77 and 85) on *X. schlechteri* refer to it as *Xerophyta viscosa*. This has been taken into account in our dataset. To understand the geographical distribution of focal species relative to research efforts, we categorized studies into three groups: (i) native—conducted by researchers working within the native range of the focal species, (ii) collaborative—conducted by researchers working both within and beyond the native range of the focal species, and (iii) international—conducted by researchers working outside of the focal species native range. To understand research methodologies, we classified studies into six categories: (i) studies involving analysis of genes, their function and expression using different techniques were assigned to genomic studies; (ii) articles that were investigating ribonucleic acid (RNA) transcripts were classified as transcriptomic studies; (iii) articles that reported the role of proteins were categorized as proteomic studies; (iv) studies that involved profiling and quantification of primary and secondary metabolites were captured as metabolomic studies; (v) studies involving the analysis of microbes associated with resurrection plants were classified as metagenomics; and (vi) physiological studies included studies looking at a range of responses from RWC, dry mass, germination, stomatal conductance, photosynthesis rate, antioxidant enzyme activity, to ultrastructural image–based technologies.

Data were extracted and arranged in Microsoft Excel 2019 and analyzed in R version 1.2.5033 (R Studio Inc., Boston, MA, USA, 2019). We computed the number of studies conducted on each species annually, if they were native, collaborative or international study, and what the methodologies were. The R Packages gplots, ggplot2 and plotly were used for data visualization.

## 3. Results

After a rigorous screening and filtering, the literature search yielded 185 articles (Figure 1). We used these articles to identify the best studied resurrection plant species, to summarize the geographical distribution of focal species relative to research efforts, and to quantify the research methodologies used. A total of 15 angiosperm resurrection plants were selected for inclusion, namely *B. hygrometrica*, *C. plantagineum*, *C. pumilum*, *C. wilmsii*, *E. nindensis*, *H. rhodopensis*, *L. brevidens*, *M. flabellifolia*, *O. thomaeum*, *R. nathaliae*, *R. serbica*, *S. stapfianus*, *T. loliiformis*, *X. humilis*, and *X. schlechteri*.

### 3.1. Well Studied Angiosperm Resurrection Plants

**Figure 2:**
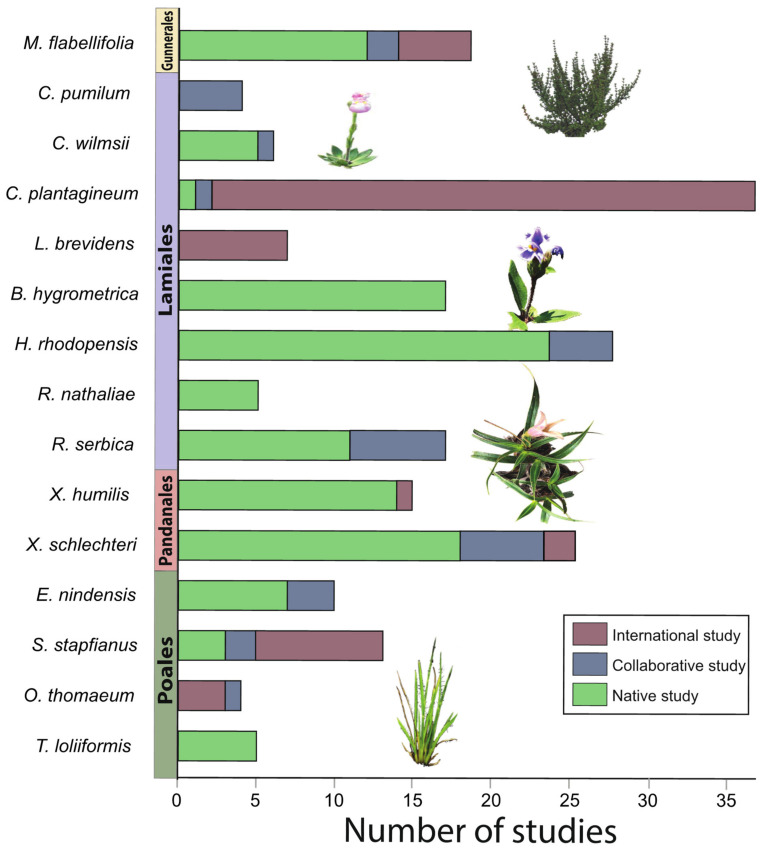
The number of studies published on each resurrection plant species. Species are ordered phylogenetically. Studies are categorized as either native (conducted by researchers working in the native range of the species), collaborative (involving researchers from both within and beyond the native range of the species) or international (studies conducted by a team working outside of the native range of the species).

To identify the best studied resurrection plants, we computed the total number of studies focusing on each species. Some articles analyzed more than one resurrection species and these studies were counted for each plant species. Therefore, the sum of all studies reported on each plant (211) exceeds the total number of studies (185). The largest proportion (17.5%, 37/211) of studies were conducted on *C. plantagineum*. Following that, 13.7% of studies were conducted on *H. rhodopensis*, 11.9% on *X. schlechteri*, 8.5% on *M. flabellifolia*, 8.1% on *B. hygrometrica*, 7.1% on *X. humilis*, 7.6% on *R. serbica*, 6.2% on *S. stapfianus*, 4.7% on *E. nindensis*, 3.3% on *L. brevidens*, 2.8% on *C. wilmsii*, 2.4% on *R. nathaliae*, and *T. loliiformis*, and 1.9% on *C. pumilum* and *O. thomaeum* (see Figure 2).

### 3.2. Distribution of Resurrection Plants

**Figure 3:**
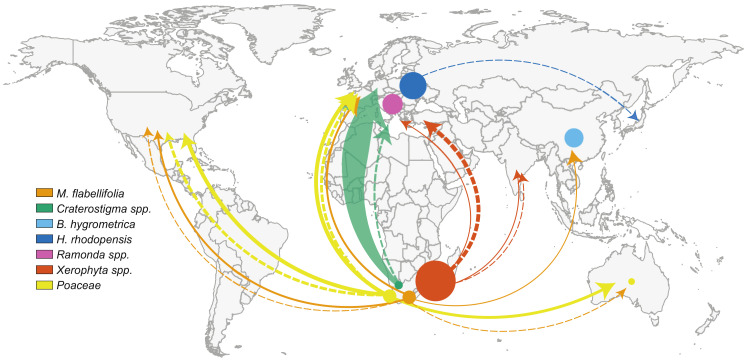
Map showing where the model resurrection plants are native to relative to where they have been studied. Circles indicate the native area of the species and are scaled by the number of studies conducted in their native range. Arrows point to the location where international and collaborative studies have been conducted and are scaled by the number of studies. Dashed arrows are for collaborative studies and solid arrows are for international studies. Plants in the same genus (or family for grasses) are consolidated for simplicity and studies are grouped by continent.

Resurrection plants occur and have been studied across the globe, from Africa to Asia, Oceania, Europe, and North America (Table 1). Researchers working in South Africa have published more studies (56) on resurrection plants than any other nation. These studies investigated the focal species *M. flabellifolia* (12), *Craterostigma* spp. (3), *Xerophyta* spp. (39) and multiple species within *Poaceae* (10). Bulgaria was the country with the second most studies—primarily focused on *H. rhodopensis* (24) and *R. serbica* (1), followed by China with 17 studies of *B. hygrometrica* (Figure 3).

Most studies (55.1%, 102/185) were conducted by teams working within the native range of the focal species (Figure 2). Six countries explored their native resurrection plants, namely Australia, Bulgaria, China, Kosovo, Serbia, and South Africa. Other studies (30.8%, 57/185) were conducted by teams working outside the native range of the focal species. These international studies were distributed across the world, with major contributions from European countries, including Germany, Hungary and Italy (Figure 3). Other international studies were conducted in China, India, Kenya, and the United States of America (USA). Germany, in particular, studied many non–native resurrection species including *Craterostigma* spp. (35), *L. brevidens* (6), *M. flabellifolia* (2) and grasses in *Poaceae* (2). We also identified a number of collaborative studies (14%, 26/185) involving scientists working both within and beyond the native range of the focal species. Many of these involved participants from Asia, Europe, and USA who established collaborations with researchers in South Africa (Figure 3).

### 3.3. Publication Trends and Methodologies of Investigation

**Figure 4:**
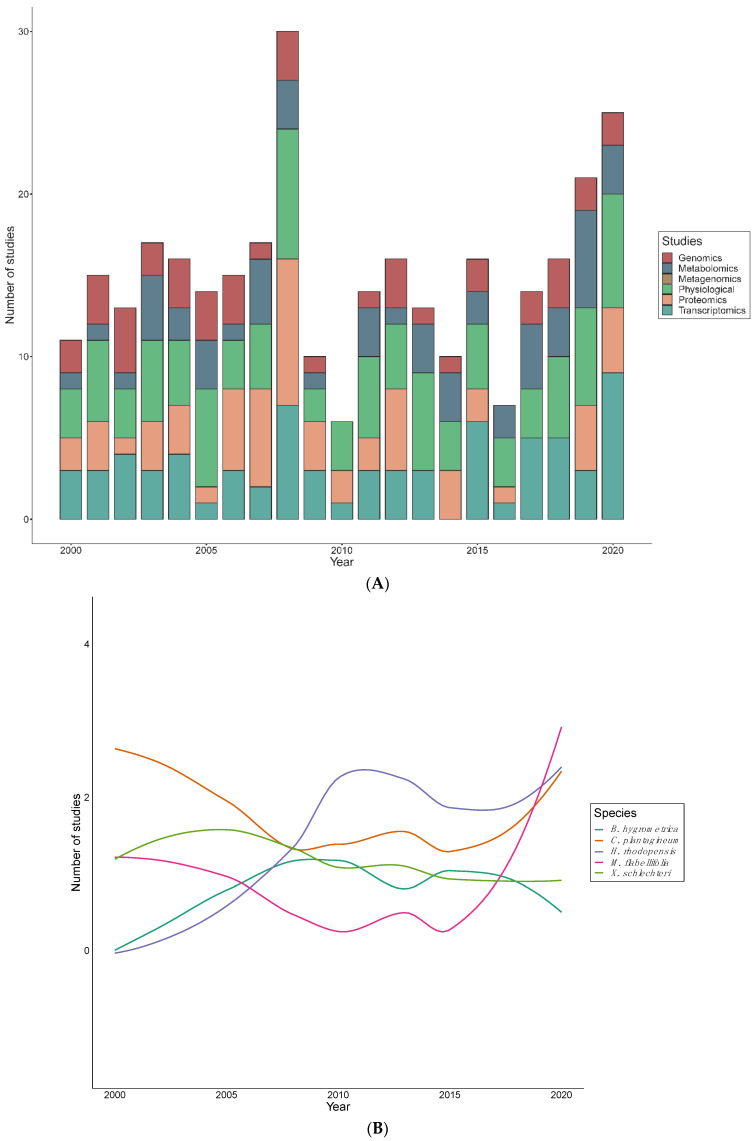
The number of studies exploring desiccation tolerance mechanisms of resurrection plants in the past two decades using various techniques. (**A**) Research articles published per year from 2000–2020 focused on Genomic (genetics), Transcriptomic (RNA), Proteomic (proteins), Metabolomic (metabolites), Metagenomic (microbes), and Physiological (biochemical) techniques to understand the mechanisms of desiccation tolerance in resurrection plants. (**B**) Analysis of publication trends of five most widely analyzed angiosperm resurrection plants, namely *B. hygrometrica*, *C. plantagineum*, *H. rhodopensis*, *M. flabellifolia* and *X. schlechteri* studies per year.

Publication rates have been relatively erratic, with 2008, and 2018–2020 showing the highest numbers of publications (Figure 4A). An average of seven articles were published per year, despite the decline in the years 2009, 2010 and 2016. In the past two decades, *C. plantagineum* was most intensively studied from 2000 and 2005, while the investigation of *H. rhodopensis* and *M. flabellifolia* took an upsurge in 2009 and 2019, respectively (Figure 4B). In contrast, *X. schlechteri* studies remained steady from the year 2000, but slightly decreased in 2015–2018. A wide range of high–throughput technologies have been employed in order to explore desiccation tolerance mechanisms in resurrection plants (Table 2). These methodologies have been applied to individual species in differing proportions (Figure 4A). Physiological studies were the most common approach to studying resurrection plants while genomic studies are the least reported. Proteomic and transcriptomic studies followed a comparable publication pattern, however, there was a significant increase of transcriptomics in 2020. Metabolomic studies have fluctuated between 2000 to 2020, however, a significant reduction occurred in 2009, 2010, and 2016. Out of 185 records, there was only one metagenome study recovered–conducted on *Ramonda* species.

## 4. Discussion

Resurrection plants are a distinctive group of species that could provide scientists with key insights into the mechanisms of extreme stress tolerance, which may ultimately be leveraged to improve tolerance in drought–sensitive crops. It is not surprising that resurrection plants have received increasing research attention over the past years as appreciation for their phenomenal defense mechanisms has become more widespread. Our study was based on the analysis of 185 empirical studies of angiosperm resurrection plants published over the past two decades. Our systematic review identifies the best studied resurrection plants, the geographical distribution of species and research efforts across the world, and changes in the methodologies used for investigation.

Our findings revealed that out of the 15 model species, *C. plantagineum* was the most studied, with 17.5% of all publications in the past 20 years devoted to this species. The publication trends of *C. plantagineum* were at the maximum between 2000 and 2005. However, in some studies *C. plantagineum* was explored concurrently with other resurrection plants. For instance, Moore et al. [29] simultaneously investigated the functional role of arabinose polymers as plasticizers and maintaining flexibility in the cell wall of *C. plantagineum*, *M. flabellifolia*, *E. nindensis* and *Xerophyta* spp. These multi–species approaches enable researchers to identify conserved aspects of desiccation tolerance across disparate clades. Surprisingly, 95% studies of *C. plantagineum* were international studies, with German researchers leading the work on this species, although it is native to Southern Africa. Another resurrection species, *H. rhodopensis*, was extensively studied with 13.7% of publications addressing desiccation tolerance mechanisms in this species. The desiccation tolerance features of *H. rhodopensis* have been recently reviewed by Liu et al. [12]. Interestingly, *H. rhodopensis* was also frequently investigated alongside other species. For example, a study by Vassileva et al. [62] assessed leaf micromorphology of both *H. rhodopensis* and *R. serbica*, and both species are native to Bulgaria. *Xerophyta* spp. were also widely explored, constituting 19% of all studies. Although *C. pumilum*, *L. brevidens*, *O. thomaeum*, *R. nathaliae*, and *T. loliiformis* have been classified as best studied resurrection species by others, our findings showed that these were among the least studied species in our dataset. This is partially explained by the fact that some of the work on *O. thomaeum*, is not related to desiccation tolerance mechanisms and was therefore not included in the current analyses [31,63]. Despite the relatively low number of studies on *O. thomaeum*, there is a chromosome-level genome assembly and associated RNA-seq data [16,20] for the species. This is in contrast to some of the other species, such as *R. nathaliae*, for which there are no genomic resources available. We suggest that more research is required to understand how these species respond to desiccation and to identify mechanistic deviations relative to other angiosperms. The families of *Poaceae* and *Cyperaceae* contain many of the known desiccation tolerant species [64], however, within those families only few species have been extensively studied. Despite some similarities among angiosperm resurrection plants, substantial variations in mechanisms of tolerance have been identified [5,65], including differences in the production of metabolites, expression of genes, synthesis of proteins and physical and ultrastructural features. Thus, there is still room to explore the intricate and diverse mechanisms of stress tolerance exhibited by these species.

We also investigated the distribution of resurrection plant research across the world. Resurrection plants are common in arid tropical and subtropical regions, occurring mostly on mountains with exposed and rocky surfaces [9,66]. Our results showed that most studies of resurrection plants were conducted within the native range of the species, likely because access to plant material is much easier when working within the native range of the focal species. In particular, most studies were executed in South Africa which is likely due to the remarkable diversity of resurrection plants native to Southern Africa. In fact, seven out of the fifteen best studied species are native to Southern Africa, which is known as an epicenter of diversity of resurrection plants [64,65,67,68]. Despite the species richness in Africa, resurrection plant research still lags behind in other African countries, possibly due to lack of resources or funding. Outside of Africa, Bulgaria has been a major contributor to resurrection plant research with many studies of *H. rhodopensis* and *R. serbica*, both of which are native to the Balkan and Rhodope mountains of Eastern Europe [6,69]. Similarly, 99% of the studies conducted on *B. hygrometrica* were carried out within the species native range of China. It is noteworthy that limited collaborative studies were discovered. A number of the resurrection plants native to the Global South were studied in European countries without instigating collaboration. This could be attributed to the narrow distribution of resurrection plants in the Global North as well as differences in funding and resources available for research in the Global North relative to the Global South [70]. Despite these imbalances, South Africa remains a leader in the research of angiosperm resurrection plants. That being said, multiple collaborative efforts were identified linking researchers in South Africa to international scientists in Asia, Europe, and North America. These collaborative efforts may facilitate the exploration of desiccation tolerance mechanisms and minimize costs for those residing in indigent countries.

High–throughput methodologies have been increasingly applied to resurrection plants and provide comprehensive insight into the mechanisms of desiccation tolerance. We found that a greater proportion of studies focused on physiological and transcriptomic techniques, relative to other methodologies. The physiological studies correlate with average research publications per year. This could be attributed to the fact that physiological studies typically do not involve advanced technologies compared to other methodologies and are therefore easier to initiate. For instance, light microscope, scanning electron microscope (SEM), and transmission electron microscopes were used to examine physiological [68,71] and ultrastructural [24,65,72] aspects of resurrection plants.

In the past years, scientists have explored resurrection plant genomes, yet only 5 of the 15 best studied resurrection plants have a publicly available whole genome assembly [73]. Although genome sequencing of desiccation tolerant plants has sought to identify a “footprint” of vegetative desiccation tolerance in these species [3,74], such signatures have not been forthcoming. There have been some genetic studies of *Ramonda* spp. [75], however, the focus was not directed to the functional role of genes under desiccation conditions. There are various emerging genetic technologies such as genome-editing (clustered regularly interspaced short palindromic repeats) applied for gene identification in microorganisms and plant species, but this approach is rarely employed in resurrection plant research. However, Costa et al. [76] suggested a futuristic approach of further studying desiccation tolerance genes by breeding plants with improved tolerance to drought. Nevertheless, Hilhorst et al. [11] postulated that more genomic research is anticipated in the near future.

The rapid increase in omics research in recent years correlates with the advances in technologies. It is worth noting that high–throughput technologies are expensive and mostly performed in well–developed countries, while lower income countries lag behind. Our analyses show that most expensive high–throughput technologies were applied in studies conducted in the USA, Australia, China, and European countries. For instance, all genome sequencing studies have emanated from the Global North, with the exception of China–a notable outlier relative to other countries in the Global South. Transcriptome studies were also predominately conducted in the Global North including those on *B. hygrometrica* [21], *C. plantagineum* [77], *L. brevidens* [20], *E. nindensis* [19], *O. thomaeum* [16] and *S. stapfianus* [34]. Similarly, proteome studies were mostly performed in the Global North on *C. plantagineum* [77], *R. serbica* and *B. hygrometrica* [78], although two papers on *X. viscosa* [51,79] have come from South Africa. Metabolomic studies, on the other hand show equal distribution been the Global North, on *S. stapfianus* [34], *C. plantagineum* [77], *H. rhodopensis* [80] and *B. hygrometrica* [81], and the Global South on, *M. flabellifolia* [82] and *X. schlechteri* [24]. In addition, the execution of these high–throughput techniques was also noted in collaborative studies between well resourced (mostly Northern) and under resourced (mostly Southern) countries, such as those by Plancot et al. [55], Costa et al. [16], Vidović et al. [83] and Pardo et al. [19]. These patterns further reinforce the notion that economic barriers limit participation in omics studies by teams working exclusively in the Global South. Omics research is underrepresented in Africa, not only in plant biology, but also in the biomedical research [70] and these challenges are mainly associated with socio–economic factors. In this regard, we urge researchers to develop more collaborations between countries with a rich diversity of desiccation tolerant plants and countries with economic resources. Such collaborations will not only aid in understanding desiccation tolerance and facilitating the biotechnological roll out of drought tolerant crops, but will also expand participation and bring diverse expertise to resurrection plant research. This is critical for food security especially in economically depressed African nations, where 95% of agriculture relies on rainfall and where, due to climate change, droughts are predicted to have become so severe that by 2050 all conventional farming practices will be abandoned [84,85].

Collectively, molecular studies have revealed that there are core transcripts, proteins and metabolites produced by different resurrection plants. However, there are also considerable mechanistic differences among genera and species [12,74]. Thus, it is imperative to continue in depth systems studies on as many resurrection plants as possible to fully understand the spectrum of ‘’tools” used, and the manner in use thereof, in achieving desiccation tolerance. With this insight, the use of individual resurrection species as models for specific crops becomes more feasible. Interestingly, some individual species show natural variation and plasticity in desiccation tolerance. Bentley et al. [82] reported differences in the metabolites of *M. flabellifolia* from different geographical regions. This could be due to numerous environmental factors including the soil/bedrock type and respective microbial interactions, light intensities, temperatures, rainfall and biotic stresses experienced in different regions. The nature of such fine tuning could explain “leeways” in vegetative desiccation tolerance and is of interest to the medical and cosmetic industries. *M. flabellifolia* itself has several medicinal [82] and cosmetic [86] applications. We suggest that investigation of a species across its geographic range could provide insight on the genetics, metabolites, microbes, and environmental factors that impact desiccation tolerance.

The role of roots in desiccation tolerance is largely unexplored. The few studies that report on roots include a physiological study on roots of *X. schlechteri* [87] and *H. rhodopensis* [88], one phenology and carbohydrate metabolism in *C. plantagineum* [89], one metabolomic and transcriptomic study in *T. loliiformis* [15] and one study on the significant role of phytohormones in dehydrated *H. rhodopensis* [90]. While diverse in nature, these studies confirm the central role of sucrose and antioxidants in desiccation tolerance, but also reveal stark differences in root longevity. Roots of *C. plantagineum* senesce after rehydration and new ones are generated [89]. In the other two species, roots are maintained, and indeed senescence is actively suppressed in *T. lolliformis* [15]. Numerous questions remain and we propose that root systems studies are sorely needed.

Similarly, there has been limited research published on the microbiome associated with resurrection plants. The only metagenomic study recovered from all 185 records in our analyses explored rhizospheric bacterial diversity associated with *Ramonda* species [91]. Rakic et al. [8] also investigated the role of mycorrhizal fungi associated with *R. nathaliae* roots in facilitating mineral stress associated with serpentine soils, but this was not directly related to their desiccation tolerance.

As indicated before, plant–microbiome interactions play an important role in alleviating environmental stress, improving nutrient uptake and facilitating plant growth [25]. Therefore, metagenomics studies aimed at the identification and characterization of microbiota inhabiting the rhizosphere and roots of resurrection plants could provide insight into the functional role of plant microbiomes and their influence on desiccation tolerance mechanisms. Further investigation should be conducted to understand the role of plant associated microbiota under desiccating conditions.

## 5. Conclusions and Future Research

Resurrection plants are excellent models for investigating plant responses to environmental stresses and adaptive mechanisms of water stress tolerance. Our analyses showed that research efforts have been skewed to *C. plantagineum*, *H. rhodopensis*, *B. hygrometrica*, and *Xerophyta* spp. We also identified a link between the geographical range of the focal resurrection plants and the national affiliations of the researchers studying them, with most resurrection species being studied within their native range. The integration of multi–omics studies provide new opportunities to understand desiccation tolerance mechanisms. Despite extensive research on the intricate mechanisms of vegetative desiccation tolerance, our results showed that the biggest gaps lie belowground, and more research is needed to understand root molecular physiology, metagenomics and plant microbe interactions.

This systematic study identifies knowledge gaps pertaining to resurrection plants and points towards socio–economic barriers impacting research outputs. We recommend that (i) despite the available literature on the well-studied angiosperm resurrection plants (*O. thomaeum*, *S. stapfianus*, *T. loliiformis*, *X. humilis*, *C. pumilum*, *C. wilmsii*, *L. brevidens*, *R. serbica*, and *R. nathaliae*) more research is still needed on these species; (ii) researchers establish collaborations across geographic and socio economic space to apply new methodologies to resurrection plants native to Global South; (iii) studies aim to identify the complex interplay between, and regulatory features associated with, the genome, transcriptome, proteome, metabolome and consequent physiological outplay; and. Within these, there are still big gaps in our knowledge. For example, there are no studies on epigenetic regulation of vegetative desiccation tolerance, although some hint that this plays a significant role (reviewed in [92]); (iv) that more focus should be given to studies aimed at exploring the belowground dynamics of desiccation tolerance.

## Figures and Tables

**Table 1:** The best studied angiosperm resurrection plants in the past two decades and their native range.

Resurrection Plant	Continent	Region	Reference
Monocot			
*E. nindensis*	Africa	Southern Africa	[29,30]
*O. thomaeum*	Asia and East Africa	India, Northeast tropical and East tropical Africa	[31]
*S. stapfianus*	Africa	South Africa	[32,33,34]
*T. loliioformis*	Australia	Australia	[15]
*X. humilis*	Africa	Southern Africa	[29,35]
*X. schlechteri*	Africa	Lesotho, South Africa, Swaziland	[10,24,29]
Dicot			
*B. hygrometrica*	Asia	China	[7]
*C. plantagineum*	Africa	Kenya, South Africa	[36]
*C. pumilum*	Africa	East Africa	[37]
*C. wilmsii*	Africa	South Africa	[38]
*H. rhodopensis*	Europe	Bulgaria	[6]
*L. brevidens*	Africa	Kenya	[39]
*M. flabellifolia*	Africa	Namibia, South Africa, Zimbabwe	[40]
*R. nathaliae*	Europe	Serbia, Bulgaria	[41]
*R. serbica*	Europe	Serbia, Bulgaria	[8]

**Table 2:** Tools and technologies used for investigation of desiccation tolerance mechanisms in resurrection plants.

Technology–Based Approach	Genomics	Transcriptomics	Proteomics	Metabolomics	Physiology	Metagenomics
	DNA sequencing, genetic profile, genetic mapping, structural & functional genomics	RNA sequencing, expression profiling, transcriptional regulation	Protein identification, quantification, Translation modification	Metabolites profiling, identification & quantification	Morphological, biochemical & phenotypical characterization	Bacterial and fungal and viral nucleic acids analysis
Methodology and Quantitative techniques	Southern blotting DNA sequencing and cloning Polymerase chain reaction (PCR) DNA microarray	RNA gel blot (Northern blotting) cDNA-AFLP qRT-PCR	Western blotting 1/2D SDS–PAGE Protein microarray iTRAQ proteomic analysis	Sonication Chromatography-based techniques	Machine-learning Digital colour camera (JVC) Light microscope Confocal laser scanning microscope	Nucleic acid extraction Denaturing gradient gel electrophoresis (DGGE) PCR
High–throughput techniques	Single nucleotide polymorphism (SNP) Marker-assisted selection Quantitative trait loci (QTL) mapping	Hybridization technology based chip (cDNA-chip), Serial analysis of gene expression (SAGE) Expressed sequence tags (ESTs) RNA-sequencing, RNA-PET-seq sRNA-seq	Matrix assisted laser desorption ionization (MALDI-TOF/MS) LC-MS/MS X-ray crystallography NMR spectroscopy	Ion mobility spectrometry-mass spectrometry (IM-MS) NMR spectroscopy Gas chromatography-mass spectrometry (GC-MS), LC-MS/MS Electrospray ionization multistage tandem mass spectrometry (ESI-MS)	X-ray tomography Transmission electromagnetic microscope Scanning electron microscope Fluorescence and infrared imaging Spectral, 3D, magnetic resonance imager	Whole metagenomic shotgun sequencing Amplicon metagenomic sequencing
References	[42,43,44,45]	[15,46,47,48,49,50]	[51,52,53]	[23,54,55]	[24,39,56,57]	[58,59,60,61]

Key: cDNA-AFLP (cDNA-amplified fragment length polymorphism); qRT-PCR (real-time quantitative PCR); RNA-PET-seq (paired end sequencing); sRNA-seq (small RNA sequencing); SDS-PAGE (sodium dodecyl sulphate–polyacrylamide gel electrophoresis); iTRAQ (isobaric tag for relative and absolute quantitation); LC-MS (liquid chromatography-mass spectrometry); NMR (nuclear magnetic resonance).

## Data Availability

Data used in this study are available in the Supplementary Materials.

## Supplementary Materials

The following are available online at https://www.mdpi.com/article/10.3390/plants10122784/s1, Table S1. A list of articles used in this study as well as captured data.
